# Prevention of Prostate Cancer with Oleanane Synthetic Triterpenoid CDDO-Me in the TRAMP Mouse Model of Prostate Cancer

**DOI:** 10.3390/cancers3033353

**Published:** 2011-08-19

**Authors:** Xiaohua Gao, Dorrah Deeb, Yongbo Liu, Ali S. Arbab, George W. Divine, Scott A. Dulchavsky, Subhash C. Gautam

**Affiliations:** 1 Department of General Surgery, Henry Ford Health System, Detroit, MI 48150, USA; E-Mails: tgao1@hfhs.org (X.G.); ddeeb1@hfhs.org (D.D.); bliu1@hfhs.org (Y.L.); sdulcha1@hfhs.org (S.A.D.); 2 Department of Diagnostic Radiology, Henry Ford Health System, Detroit, MI 48150, USA; E-Mail: sali1@hfhs.org; 3 Department of Public Health Sciences, Henry Ford Health System, Detroit, MI 48150, USA; E-Mail: gdivine1@hfhs.org

**Keywords:** prostate cancer, chemoprevention, CDDO-Me, apoptosis, Akt/mTOR/NF-κB signaling

## Abstract

2-Cyano-3,12-dioxooleana-1,9(11)-dien-28-oic acid (CDDO), a synthetic analog of oleanolic acid, and its C28 methyl ester derivative (CDDO-Me), have shown potent antitumorigenic activity against a wide range of cancer cell lines, including prostate cancer cells *in vitro*, and inhibited the development of liver and lung cancer *in vivo*. In the present study, we examined the efficacy of CDDO-Me in preventing the development and progression of prostate cancer in the transgenic adenocarinoma of the mouse prostate (TRAMP) model. CDDO-Me inhibited the growth of murine TRAMPC-1 prostate cancer cells by inducing apoptosis through the inhibition of antiapoptotic p-Akt, p-mTOR and NF-κB. Early intervention with CDDO-Me (7.5 mg/kg) initiated at five weeks of age for 20 wk inhibited the progression of the preneoplastic lesions (low-grade PIN and high-grade-PIN) to adenocarcinoma in the dorsolateral prostate (DLP) and ventral prostate (VP) lobes of TRAMP mice. Even delayed administration of CDDO-Me started at 12 wk of age for 12 wk inhibited the development of adenocarcimona of the prostate. Both early and late treatment with CDDO-Me inhibited the metastasis of tumor to the distant organs. Treatment with CDDO-Me inhibited the expression of prosurvival p-Akt and NF-κB in the prostate and knocking-down Akt in TRAMPC-1 tumor cells sensitized them to CDDO-Me. These findings indicated that Akt is a target for apoptoxicity in TRAMPC-1 cells *in vitro* and potentially a target of CDDO-Me for inhibition of prostate cancer *in vivo*.

## 1. Introduction

Carcinoma of the prostate (CaP) is the most commonly diagnosed cancer and second leading cause of cancer-related mortality in men in the United States. Current therapies (radical prostatectomy, local radiotherapy or brachytherapy), while successful for treating localized prostate cancer, are of limited efficacy against metastatic disease [1,2]. Epidemiological studies have shown that high-fat diet and lifestyle are prominent risk factors for prostate cancer [3]. In contrast to the high incidence of prostate cancer in North America and other high-fat diet-consuming countries of the Western Hemisphere, the incidence of prostate cancer is very low in Asian men. Low incidence of prostate cancer among Asian men has been attributed to the consumption of low-fat diet and high intake of dark green vegetables, fruits, and soy products [4]. The cancer-preventing effects of plant-derived foods are due to the presence of polyphenolic phytochemicals with strong antioxidant and anti-inflammatory activity [5]. Indeed, the activity of plant-derived polyphenolic compounds in preventing and/or slowing the progression of prostate cancer has been demonstrated in animal studies and clinical trials [6-8]. Because the incidence of CaP increases with advancing age and the multistep oncogenic process leading to CaP is completed over decades, early intervention with natural compounds or their synthetic analogs with proven anti-inflammatory and antioxidant activity represents a promising approach to preventing/delaying the incidence, progression, recurrence, morbidity and mortality associated with the cancer of prostate.

Oleanolic acid and ursolic acid are naturally occurring triterpenoids that have been used in traditional medicine as anti-cancer and anti-inflammatory agents [9-11]. Recent studies have shown that the synthetic derivatives of oleanolic acid such as 2-cyano-3,12-dioxooleana-1,9(11)-dien-28-oic acid (CDDO) and its methyl ester (CDDO-Me) or imidazole (CDDO-Im) derivatives exhibit greater anti-inflammatory and antitumorigenic activity than oleanolic acid itself [12-14]. Synthetic CDDOs have shown potent antiproliferative and antitumorigenic activity against diverse types of tumor cell lines, including leukemia, multiple myeloma, osteosarcoma, breast, brain, prostate and lung cancer cell lines [15-19]. Although the mechanisms of the anticancer effects of CDDOs are not fully understood, cancer cell differentiation, apoptosis and modulation of MAPK (Erk1/2), NF-κexhibited moderately differentiated (MD) adenocarcinoma B, TGF-β/Smad and PPARγ signaling pathways contribute to the antitumor activity of CDDOs [18,20-24]. CDDOs have also shown chemopreventive activity in animal models of liver, breast and lung cancer [25-27]. We have previously shown that CDDOs inhibit the growth of hormone-sensitive and hormone-refractory prostate cancer cell lines *in vitro* and *in vivo* by inducing apoptosis [16,28]. Furthermore, CDDO, the parent compound delayed the progression of prostate cancer in TRAMP mice [29].

Our previous *in vitro* studies showed that CDDO-Me has more potent antiproliferative and antitumorigenic activity against prostate and other cancer cell lines than CDDO [16,30,31]. In the present study, we investigated CDDO-Me for prevention of the development and progression of CaP in the TRAMP mouse model of prostate cancer. Results showed that treatment with CDDO-Me inhibits the progression of preneoplastic lesions to adenocarcinoma and metastasis of prostate cancer to the distant organs. The tumor inhibitory effect of CDDO-Me was associated with the inhibition of prosurvival p-Akt, NF-κB and p-mTOR signaling molecules.

## 2. Results

### 2.1. CDDO-Me Inhibits Proliferation and Induces Apoptosis in TRAMPC-1 Prostate Carcinoma Cells

**Figure 1.**
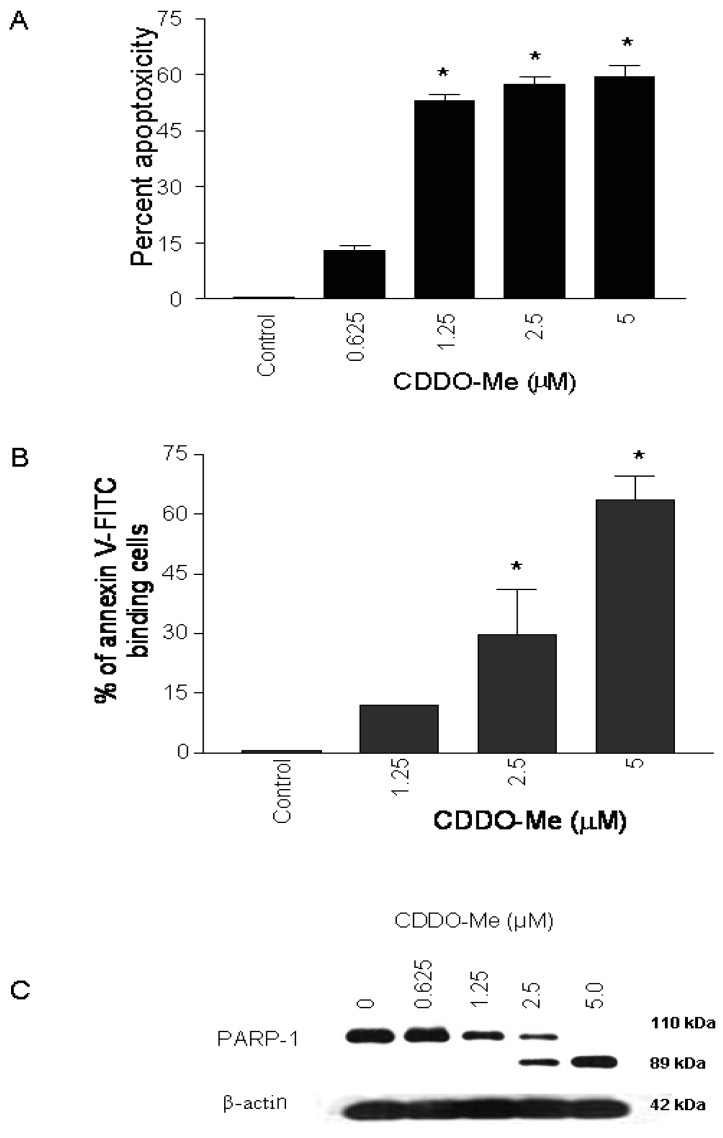
CDDO-Me inhibits growth and induces apoptosis in TRAMPC-1 prostate cancer cells. **(A)**. Effect on cell viability. 1 × 10^4^ TRAMPC-1 cells were treated with CDDO-Me at concentrations ranging from 0 to 5 μM for 72 h in triplicate in 96-well microtiter plate. Cell viability was measured by MTS assay using CellTiter AQueous assay system from Promega and from decrease in viability cytotoxicity (apoptoxicity) was determined; **(B)** & **(C)**. CDDO-Me induces apoptosis in TRAMPC-1 cells. TRAMPC-1 cells were treated with CDDO-Me for 24 h at concentrations as shown and binding of annexin V-FITC (B) and cleavage of PARP-1 (C) was measured by flow cytometry and western blotting, respectively. *Significantly different from control (*P* < 0.05).

To test the effect of CDDO-Me on proliferation of prostate cancer cells, 1 × 10^4^ TRAMPC-1 cells were plated in 96-well microtiter plates for 24 h and then treated with CDDO-Me for 72 h at concentrations ranging from 0.625 to 5 μM. Viability of cultures was determined by MTS assay and percent cytotoxicity (apoptoxicity) calculated from the reduction in viability of cultures. As shown in Figure 1A, significant apoptoxicity was observed in cells treated with CDDO-Me at concentrations of 1.25 μM to 5 μM (53% to 60%), which increased to nearly 80% at 10 μM (not shown).

To determine whether inhibition of proliferation of TRAMPC-1 cells by CDDO-Me was associated with induction of apoptosis, binding of annexin V to cells treated with CDDO-Me was measured. For this, TRAMPC-1 cells were treated or not with CDDO-Me (1.25 to 5 μM) for 24 h and analyzed for annexin V-FITC binding by flow cytometry. As shown in Figure 1B, the percentage of annexin V-FITC binding TRAMPC-1 cells increased in a dose-dependent manner following treatment with CDDO-Me at 1.25, 2.5 and 5.0 μM (12%, 26 % and 63%, respectively). The induction of apoptosis by CDDO-Me was confirmed by the cleavage of native PARP-1 in TRAMPC-1 cells treated with CDDO-Me (Figure 1C). Cells treated with CDDO-Me clearly showed the presence of the 89 kDa cleaved PARP-1 fragment. Together, these results demonstrated that CDDO-Me inhibits proliferation and induces apoptosis in TRAMPC-1 prostate adenocarcinoma cells.

### 2.2. CDDO-Me Inhibits Akt (p-Akt) mTOR (p-mTOR) and NF-κB Signaling Proteins in TRAMPC-1 Cells

**Figure 2.**
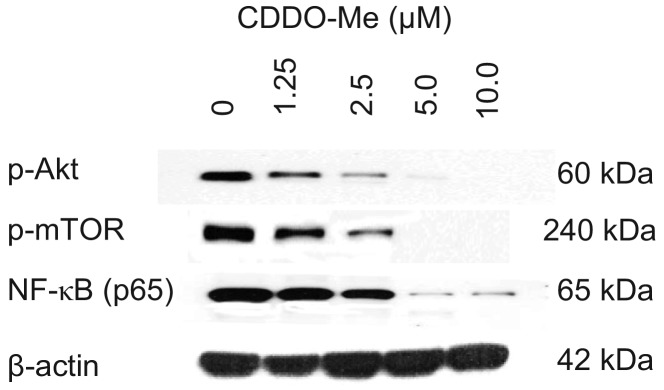
CDDO-Me inhibits antiapoptotic p-Akt, p-mTOR and NF-κB in TRAMPC-1 cells. TRAMPC-1 cells were treated with CDDO-Me (0–10 μM) for 24 h and levels of p-Akt, p-mTOR and NF-κB (p65) were analyzed by Western blotting.

Akt, mTOR and NF-κB are anti-apoptotic (prosurvival) signaling proteins that are constitutively active in a variety of human and animal cancers and provide survival advantage to cancer cells. We investigated whether TRAMPC-1 cells express constitutively active Akt (p-Akt), mTOR (p-mTOR) and NF-κB (p-65) and the effect CDDO-Me has on the expression of these signaling proteins. Cell lysates were prepared from TRAMPC-1 cells treated or not with CDDO-Me (0 to 10 μM) for 24 h and analyzed by western blotting. Figure 2 shows that TRAMPC-1 cells express p-Akt, p-mTOR and p-65 (NF-κB) (lane 1) and CDDO-Me inhibited these activated (phosphorylated) signaling proteins in concentration-related manner. Significant to complete reduction of p-Akt and p-mTOR occurred at concentrations of 1.25 μM to 10 μM whereas NF-κB was most affected at 5 to 10 μM CDDO-Me. There was no significant change in total protein levels of Akt, mTOR of NF-κB (not shown). These data demonstrated that Akt, mTOR and NF-κB, all major prosurvival signaling proteins that are critical to the development and progression of prostatic adenocarcinoma are inhibited by CDDO-Me.

### 2.3. CDDO-Me Slows Down the Progression of Prostate Cancer in TRAMP Mice

Early intervention with CDDO-Me was started at the age of 5 weeks, at which time there is no evidence of neoplastic lesions in the prostate and continued until for 7 (short-term) or 20 weeks (long-term) at a dose of 7.5 mg/kg/day, 5 days/wk by oral gavage. CDDO-Me was well tolerated without evidence of noticeable toxicity with respect to animal appearance, behavior or change in the body weight. Body weight increased as a function of age at the same rate in both the control and treatment groups and there were no deaths in the control or treatment groups during the entire course of the experiment. In addition, microscopic examination of tissue sections of liver, kidney, lung and small intestine showed no discernable histopathological changes (not shown).

**Figure 3.**
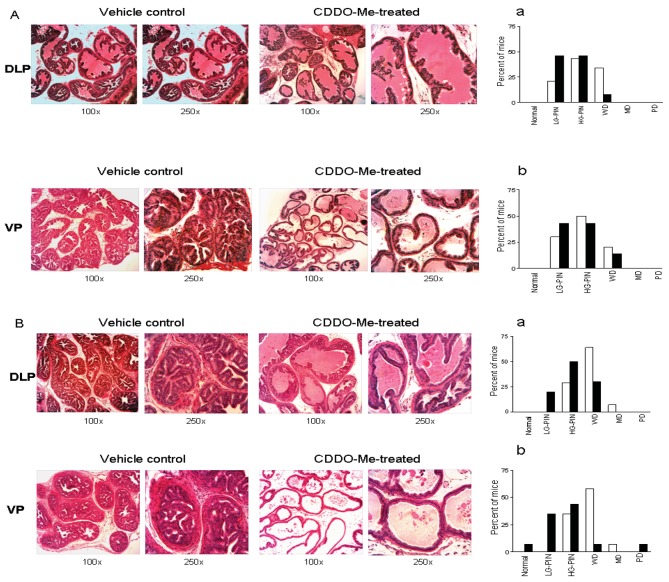
**(A)**. H&E-stained sections of DLP and VP of a 12 wk old vehicle control or CDDO-Me treated TRAMP mouse. Control and CDDO-Me treated DLP showing HG-PIN and LG-PIN lesions, respectively. Control and CDDO-Me treated VP showing well differentiated carcinoma (MD) and LG-PIN, respectively. Bar graphs show distribution of histologic grades (normal, LG-PIN, HG-PIN, WD, MD or PD carcinoma) in DLP and VP of control and CDDO-Me treated TRAMP mice (n = 14); (**B)**. H&E-stained sections of DLP and VP of a 25 wk old vehicle control or CDDO-Me treated TRAMP mouse. Control and CDDO-Me treated DLP showing well differentiated carcinoma (MD) and HG-PIN lesions, respectively. Control and CDDO-Me treated VP showing MD and LG-PIN, respectively. Bar graphs show distribution of histologic grades (normal, LG-PIN, HG-PIN, WD, MD or PD carcinoma) in DLP and VP of control and CDDO-Me treated mice (n = 14).

To determine whether CDDO-Me interferes with the development and/or progression of carcinoma of the prostate in TRAMP mice, dorso-lateral (DLP) and ventral prostate (VP) lobes of the TRAMP prostate harvested 7 wk (short-term) or 20 wk (long-term) after treatment with CDDO-Me were evaluated for the incidence and grade of tumor. Visual examination of the abdominal cavity did not reveal unusual enlargement of the seminal vesicles, prostatic lobes or pelvic lymph nodes in control or CDDO-Me treated mice. The majority of the control animals (43%) showed high-grade PIN (HG-PIN), 21% showed low-grade PIN (LG-PIN) and 34% of the animals had well differentiated carcinoma (WD) in the DLP (Figures 3A and a). The distribution of lesions in CDDO-Me treated animals was 46% LG-PIN, 46% HG-PIN and 8% WD. In VP, the distribution of pathologic lesions was very similar in both control and CDDO-Me treated mice (Figures 3A and 3b). None of the animals exhibited moderately or poorly differentiated CaP in DLP or VP in either group. Although there was a shift towards normal/non-cancerous pathological grade both in DLP and VP but it did not reach statistical significance (*P* > 0.05).

In mice treated with CDDO-Me for 20 wk**,** tumor growth in seminal vesicles was evident in 71% of control (10/14) and 14% of CDDO-Me treated mice (2/14) at necropsy. Histological evaluation of the DLP of control mice showed HG-PIN in 29% of the mice, well-differentiated adenocarcinoma in 64% of the animals and one mouse (7%) exhibited moderately differentiated (MD) adenocarcinoma (Figures 3 B, a). These data indicated that 71% of the control mice had cancerous lesions in the DLP. In contrast, DLP in 20% of CDDO-Me treated mice had LG-PIN, 50% showed HG-PIN and 30% had WD. These data demonstrated that majority of the CDDO-Me treated animals (70%) had noncancerous lesions (LG-PIN and HG-PIN) compared to 71% of the control mice that showed carcinoma of the prostate (CaP).

CDDO-Me also inhibited the progression of CaP in ventral prostate (Figure 3 B, b). 65% of the control mice had cancerous lesions (WD = 58%; MD = 7%) and 35% % had HG-PIN. Among the CDDO-Me treated animals, one mouse showed normal VP (7%), 79% had preneoplastic lesions (35% LG-PIN and 44% HG-PIN); and 7% of the mice showed WD and PD each. Both for DLP and VP, CDDO-Me treatment was associated with a shifting in pathologic grade distribution toward normal/noncancerous lesions (LG-PIN and HG-PIN, *P* < 0.01). The overall impact of long-term (20 wk) treatment with CDDO-Me on the incidence and the grade of histologic lesions in the prostate is shown in Table 1. Together, these data showed that although CDDO-Me does not prevent the development of preneoplastic lesions in majority of the animals but it significantly inhibits their progression to CaP.

### 2.4. Delayed Intervention with CDDO-Me Inhibits CaP Progression in TRAMP Mice

**Figure 4.**
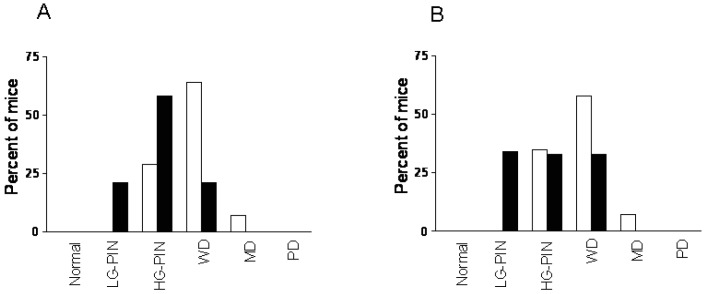
Effect of delayed treatment with CDDO-Me on tumor grade. Bar graphs show distribution of histologic grades (normal, LG-PIN, HG-PIN, WD, MD or PD carcinoma) in DLP **(A)** and VP **(B)**. Number of animals in each group = 14.

Whether CDDO-Me inhibits and/or reverses the progression of established neoplastic lesions in DLP and VP was investigated next. Treatment with CDDO-Me was delayed until mice were 12 weeks old since the majority of mice at this age exhibit HG-PIN and well-differentiated adenocarcinoma both in DLP and VP. After treatment with CDDO-Me for 12 wk DLP and VP were examined for histologic tumor grade. As shown in Figure 4A, 71% of the control mice showed cancerous lesions (64% WD and 7% MD) and 29% had HG-PIN. In contrast, only 21% of the CDDO-Me treated mice showed WD carcinoma, whereas 79% had preneoplastic lesions (21% LG-PIN and 58% HG-PIN). The distribution of lesions in VP of untreated mice was 65% cancerous (58% WD and 7% MD) and 35% preneoplastic (HG-PIN). Thirty three percent of CDDO-Me treated mice showed WD carcinoma and 66% had preneoplastic lesions (34% LG-PIN and 33% HG-PIN). These findings demonstrated that delayed treatment with CDDO-Me significantly prevent/reverse the progression of CaP in both DLP and VP glands in TRAMP mice (*P* < 0.005 and *P* < 0.03, respectively).

### 2.5. CDDO-Me Prevents Metastasis of Prostate Cancer

**Figure 5.**
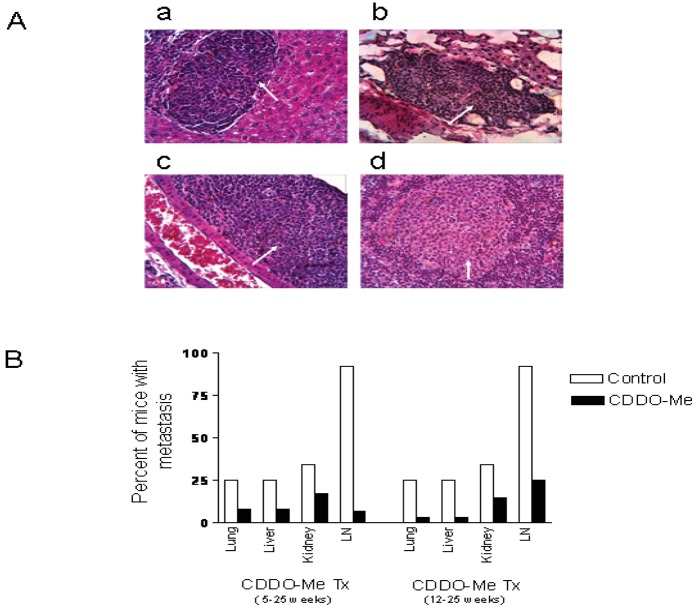
Effect of CDDO-Me on metastasis of CaP. **(A)**. H&E-stained sections of liver (a), lung (b), kidney (c) and pelvic lymph node (d) of 25 wk old control TRAMP mice showing metastatic lesions (arrows) × 250 magnification; **(B)**. Bar graph showing the incidence of metastasis in different organs in control and CDDO-Me treated TRAMP mice. The number of CDDO-Me treated mice with lymph node metastasis was significantly reduced (*P* < 0.01).

At necropsy, pelvic lymph nodes in most of the control mice were slightly enlarged compared to treated mice. No visible lesions were found in lung, liver, kidney in either group. H&E stained sections of lung, liver, kidney and pelvic lymph nodes were examined for the presence of microscopic metastases. Figure 5A shows the histological appearance of metastatic lesions in the liver (a), lung (b), kidney (c) and pelvic lymph node (d) in a control mouse. As can be seen in Figure 5B, both early (5 wk) and late age (12 wk) intervention with CDDO-Me reduced the incidence of metastasis in lung, liver, kidney and pelvic lymph nodes. The most noticeable anti-metastatic effect of CDDO-Me was observed in the pelvic lymph nodes. Twelve of the 13 control mice (92%) showed multiple metastatic foci in the lymph nodes, whereas only 2 of 12 (17%) mice in the early intervention group and 3 of 12 (25%) in the late intervention group had metastatic lesions in the lymph node. Further, the multiplicity of metastases in both treatment groups was markedly reduced compared to the control mice. Overall, these data indicated that CDDO-Me inhibits metastasis of prostate cancer to the distant organs in TRAMP mice.

### 2.6. CDDO-Me Reduces Levels of p-Akt and NF-κB (P65) in Dorsolateral Prostate

**Figure 6.**
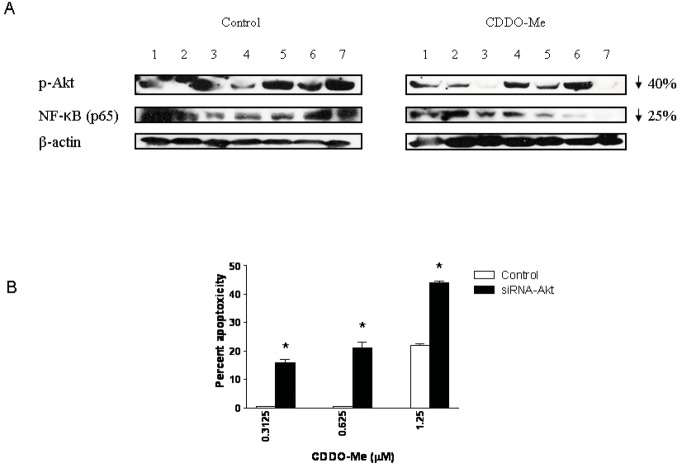
CDDO-Me inhibits p-Akt and NF-κB (p65) in DLP and knocking-down sensitizes prostate cancer cells to CDDO-Me. **(A).** DLP lysates prepared from control and CDDO-Me treated TRAMP mice were fractionated on polyacrylamide gel and p-Akt and NF-κB were detected by immunoblotting; **(B)**. TRAMPC-1 cells were transfected with double stranded siRNA-Akt as described in Materials and Methods and sensitivity of transfected cells to CDDO-Me was measured in MTS assay. ^*^Significantly different from control (*P* < 0.01).

Akt and NF-κB are major antiapoptotic molecules that confer survival advantage and resistance to anticancer therapies in cancer cells. Here we investigated whether p-Akt and NF-κB expression was altered in the DLP of mice treated with CDDO-Me for 20 wk. Figure 6A compares the levels of p-Akt and NF-κB in the DLP of control and CDDO-Me treated mice (seven each). Blot clearly shows an overall inhibition of p-Akt (40% reduction) and NF-κB (25% reduction) in the DLP of CDDO-Me treated mice compared to control mice. The levels of basal Akt or p65 were not affected by CDDO-Me (not shown).

Since Akt plays a central role in the regulation of both NF-κB and mTOR we evaluated the significance of Akt inhibition in killing of prostate cancer cells by CDDO-Me. For this, Akt was knocked-down in TRAMPC-1 tumor cells with siRNA-Akt and their response to low concentrations of CDDO-Me was measured in MTS assay. Figure 6B shows that the sensitivity of TRAMPC-1 cells to low concentrations of CDDO-Me is increased following transfection with siRNA-Akt (CDDO-Me treated cells = 16%, 21% and 44% cytotoxicity; control cells = 0%, 2% and 22% cytotoxicity at 0.325, 0.625 and 1.25 μM CDDO-Me, respectively). Minimally, increase in the sensitivity of transfected cells suggests that Akt is a good target for apoptoxicity in TRAMPC-1 cells *in vitro*, however, considering that it is also decreased in the DLP of animals treated with CDDO-Me indicates that Akt is potentially a target of CDDO-Me in prevention of prostate cancer in TRAMP mice.

### 2.7. Discussion

The present study is an extension of our previous study in which we demonstrated the efficacy of CDDO, the parent synthetic analog of oleanolic acid, in preventing the progression of preneoplastic lesions of prostate cancer in TRAMP mice [29]. Our *in vitro* studies have shown that CDDO and its methyl ester (CDDO-Me) or imidazole (CDDO-Im) potently inhibit cell proliferation and induce apoptosis in human and mouse prostate cancer cell lines [16,28,30,30]. These studies also demonstrated that CDDO was weaker than CDDO-Me or CDDO-Im in inhibiting the growth of prostate and other cancer cells in culture [16,30,31]. Since our recent study showed partial inhibition of the progression of preneoplastic lesions to CaP in TRAMP mice by CDDO [29], we proceeded to investigate the efficacy of CDDO-Me for preventing the development and progression of CaP in TRAMP mice. Oral treatment with CDDO-Me for 20 weeks was tolerated well, without toxic side effects, a prerequisite for considering new agents for human trial. Short-term early intervention (7 wk) with CDDO-Me had weak but measurable suppressive effect on the development and progression of preneoplastic LG-PIN and HG-PIN lesions in TRAMP mice. On the other hand, long-term early intervention (20 wk) with CDDO-Me showed potent inhibition of the progression of preneoplastic lesions in DLP and VP to invasive adenocarcinoma. Treatment with CDDO-Me for 20 weeks resulted in a significant shift in pathologic grade distribution toward normal/noncancerous lesions compared to the predominance of cancerous lesions in control animals. Thus, although CDDO-Me did not prevent the development of preneoplastic lesions in majority of the animals it significantly prevented their progression to the cancerous lesions. These findings are in agreement with the previously reported chemopreventive effects of CDDO analogs in aflatoxin-induced hepatic carcinogenesis and vinyl carbamate-induced lung cancer [25,27] and of herbal products such as sulforaphane, green tea polyphenols, garlic constituents and genistein on prostate tumorigenesis in TRAMP mice [6,7,32,33]. The present study showed that despite of the differences in the growth inhibitory effect of CDDO and CDDO-Me on prostate cancer cell lines *in vitro*, the chemopreventive efficacy of these synthetic oleanane analogs for CaP in the TRAMP mice is comparable. This may be due to the long-term treatment of mice with the two agents versus short term exposure of tumor cells in culture systems. Our results showed that even delayed intervention with CDDO-Me in animals with established CaP also prevented/reversed the progression of prostate cancer, suggesting therapeutic potential of CDDO-Me for prostate cancer.

Although we did not explore the mechanism of the anti-metastatic activity of CDDO-Me in this study, both early and delayed intervention with CDDO-Me reduced the incidence of metastasis of prostate cancer to liver, lung, kidney and pelvic lymph nodes. CDDO-Me was most effective in preventing metastasis of CaP to the pelvic lymph nodes. There are multiple potential targets for the antimetastatic effects of CDDO-Me, including cell adhesion molecules (E-cadherin), angiogenic growth factors and matrix metalloproteinases that remain to be explored. Because bone metastasis in TRAMP mice occurs after 30 wk of age and beyond we were not able to evaluate it in this study. It is imperative however to determine the antimetastatic activity of CDDO-Me for bone, since prostate cancer in men predominantly metastasizes to the bone.

*In vitro* studies showed that inhibition of proliferation and induction of apoptosis in TRAMPC-1 cancer cells by CDDO-Me was associated with inhibition of p-Akt, p-mTOR and NF-κB. These signaling proteins play critical role in tumor development, growth and spread by regulating cell proliferation, apoptosis, inflammation, angiogenesis, and metastasis. Activated p-Akt promotes cell survival by inactivating downstream substrates such as p-Bad, procaspase-9, and Forkhead transcription factors [34,35]. Antiapoptotic NF-κB controls the expression of genes involved in inflammation, proliferation, oncogenesis, angiogenesis, and apoptosis [36] whereas mTOR regulates cell growth, survival, ribogenesis and translation [37]. These signaling proteins are constitutively active in the DLP of the TRAMP mice and are potential targets of tumor inhibition by natural polyphenolic compounds [38]. We investigated whether CDDO-Me alters the expression of p-Akt or NF-κB (p65) in DLP in TRAMP mice. Indeed, treatment with CDDO-Me reduced the levels of both p-Akt and NF-κB (p65) in the DLP compared to control mice. Furthermore, knocking-down Akt increased the sensitivity of tumor cells to CDDO-Me, indicating that inhibition of these prosurvival (antiapoptotic) proteins is part of the mechanism by which CDDO-Me inhibits prostate tumorigenesis. This conclusion however needs more direct studies to prove the relevance of these molecules in preventing the development and progression of prostate cancer since inhibition of these signaling proteins could be independent and unrelated to the mechanisms of CDDO-Me.

In summary, we have shown the efficacy of CDDO-Me in inhibiting the progression of prostate cancer and its metastasis to the distant sites in the TRAMP mouse model of prostate tumorigenesis. Furthermore, Akt and its downstream targets such as NF-κB and mTOR appear to mediate the chemopreventive activity of CDDO-Me.

## 3. Experimental

### 3.1. Reagents and Antibodies

CDDO-Me was obtained from the Developmental Therapeutics Program, National Cancer Institute (Bethesda, MD, USA) through the Rapid Access to Intervention Development Program. Anti- p-Akt (ser^473^) antibody was from Cell Signaling Technology (Danvers, MA, USA); anti-NF-κB (p65) and anti-p-mTOR antibody was from Santa Cruz Biotechnology, Inc. (Santa Cruz, CA, USA). 96 AQueous One Solution Proliferation Assay System was from Promega (Madison, WI, USA). 100 mM stock solution of CDDO-Me was prepared in DMSO and all test concentrations were prepared by diluting the stock solution in appropriate medium.

### 3.2. Cell Line

TRAMPC-1 prostate cancer cell line derived from a primary tumor in the prostate of a TRAMP mouse was obtained from American Type Culture Collection (ATCC, Rockville, MD, USA). Cells were grown in DMEM supplemented with 4 mM L-glutamine, 0.005 mg/mL bovine insulin, 10 nM dehydroisoandrosterone, 5% fetal bovine serum, and 5% Nu-Serum IV. Cells were cultured at 37 °C in a humidified atmosphere consisting of 5% CO2 and 95% air. Cultures were maintained by subculturing cells twice a week.

### 3.3. Mice

TRAMP mice were bred by the Jackson Laboratories (Bar Harbor, ME, USA) through their Speed Expansion Service involving *in vitro* fertilization of C57BL/6 females with C57BL/6-Tg (TRAMP) 8247NG/J males. Mice were genotyped for the transgene (Tag) and delivered to us when 4 wk old. Mice were maintained in temperature-controlled room (68-72 °F) with a 12 h light/dark cycle and provided semi-purified AIN-76A mouse chow and water *ad libitum*. Mice were acclimated for one week before starting the experiment. All animal treatments were according to the protocol approved by the Institutional Animal Care and Use Committee (IACUC).

### 3.4. Treatment Protocol

Seventy five weeks old male TRAMP mice were weighed and randomized into vehicle control (n = 28), early intervention (n = 28) and delayed treatment (n= 14) groups. Mice in the vehicle control group were administered 0.1 mL of vehicle consisting of cremophor-EL:DMSO:PBS (1:1:8), 5 days a week by oral gavage for 7 or 20 weeks. In the early intervention group, mice were administered CDDO-Me at a dose of 7.5 mg/kg in 0.1 mL of vehicle, 5 days a week for 7 weeks (n = 14) or 20 weeks (n = 14). In the delayed treatment group to model the effect on already established tumors, animals were treated with CDDO-Me beginning at the age of 12 weeks through 25 weeks of age. We chose to treat animals with a dose of 7.5 mg/kg CDDO-Me since 5 to 7.5 mg/kg CDDO or CDDO-Me was found to inhibit tumor progression in TRAMP and xenograft models of CaP (28, 29). Body weight of control and CDDO-Me-treated mice was recorded each week and mice were observed for treatment related stress, such as water and food withdrawal, unusual posture, ruffled fur or listlessness. At the age of 12 weeks (short-term treatment) and 25 weeks (long-term treatment), 14 mice from each group were sacrificed 24 h after the last administration of vehicle or CDDO-Me. After opening the abdomen, mice were visually observed for the presence of tumor mass, enlargement of seminal vesicles, prostate lobes and pelvic lymph nodes. Dorso-lateral (DLP) and ventral (VP) prostatic lobes, liver, lung, kidney and pelvic lymph nodes were harvested. Tissue samples were processed for histological and biochemical analyses.

### 3.5. Pathological Grading of Lesions and Detection of Metastases

The pathologic grading of prostate cancer was according to the grading system for TRAMP described by Greenberg and colleagues [39,40]. Following this grading system, prostate lesions in the DLP and ventral prostate lobes were histologically graded as normal (ducts lined with single layer of secretory epithelial cells surrounded by 2–3 cell layers of fibromuscular stroma; low-grade PIN (epithelial cells with variably elongated nuclei with condensed chromatin); high-grade PIN (epithelial stratification and tufting, presence of micropapillary and cribiform structures); well differentiated (WD) carcinoma (epithelial cells invading fibromuscular stroma) and moderately (MD) to poorly differentiated (PD) adenocarcinoma of the prostate (sheets of neoplastic cells with little or no glandular structures). Ten randomly selected microscopic fields on H&E stained sections of the DLP and VP were scored for the incidence and the pathologic grade of the prostate cancer in control and CDDO-Me-treated TRAMP mice. For the incidence of metastasis (percentage of mice with metastatic lesions), H&E stained sections of liver, lung, kidney and pelvic lymph nodes were evaluated microscopically.

### 3.6. MTS Assay

Cells (1 × 10^4^) were seeded into each well of a 96-well plate in 100 μL of tissue culture medium. After 24 h incubation to allow cells to adhere, cultures were treated with CDDO-Me at concentrations of 0.625 μM to 5 μM for 72 h. Cell viability was then determined by the colorimetric MTS assay using CellTiter 96 AQueous One Solution Proliferation Assay System.

### 3.7. Western Blotting

TRAMPC-1 cell and DLP tissue lysates were prepared in lysis buffer (1% Triton-X 100 (v/v), 10 mM Tris-HCl (pH 7.5), 5 mM EDTA, 150 mM NaCl, 10% glycerol, 2 mM sodium vanadate, 5 μg/mL leupeptin, 1 μg/mL aprotinin, 1 μg/mL pepstatinin, and 10 μg/mL 4-2-aminoethylbenzenesulfinyl fluoride). Lysates were clarified by centrifugation at 14,000 × g for 10 min at 4 °C, and protein concentrations were determined by Bradford assay. Samples (50 μg) were boiled in an equal volume of sample buffer (20% glycerol, 4% SDS, 0.2% Bromophenol Blue, 125 mM Tris-HCl (pH 7.5), and 640 mM 2-mercaptoethanol) and separated on pre-casted Tris-glycine polyacrylamide gels using the XCell Surelock™ Mini-Cell, in Tris-Glycine SDS running buffer, all from Novex (Invitrogen, Carlsbad, CA, USA). Proteins resolved on the gels were transferred to nitrocellulose membranes. Membranes were blocked with 5% milk in 10 mM Tris-HCl (pH 8.0), 150 mM NaCl with 0.05% Tween 20 (TPBS) and probed using protein specific antibodies to p-Akt (ser^473^), NF-κB (p65), p-mTOR or β-actin (loading control) and HRP-conjugated secondary antibody. Immune complexes were visualized using chemiluminescence reagent from Thermo Fisher Scientific (Rockford, IL, USA). Protein bands were imaged and band densities analyzed using the NIH/Scion image analysis software.

### 3.8. Histology

Tissue specimens of prostate gland, liver, lung, kidney, small intestine and pelvic lymph nodes were fixed in 10% neutral buffered formalin for 48 h and then embedded in paraffin. Five micrometer thick sections were cut and stained with H&E for routine histology. Histologic interpretations of tumor grade and tumor metastasis were made in a blinded fashion.

### 3.9. siRNA Transfection

For silencing of Akt, TRAMPC-1 cells were transfected with double stranded siRNA-Akt using SignalSilence siRNA kit from Cell Signaling Technology (Beverly, MA, USA). Briefly, 10^6^ cancer cells were plated in 60 mm Petri dish for 24 h and treated with 3 mL of transfection medium containing 20 μg LipofectAMINE and 100 nM siRNA for 24 h. Gene silencing in transfected cells was confirmed by western blotting.

### 3.10. Statistical Analysis

Most outcomes for *in vitro* experiments were compared by Student's t-test. Wilcoxon rank sum tests were used for histologic grade which was scored from 1(normal) to 6 (poorly differentiated) as described above.

## 4. Conclusions

This study has demonstrated the ability of CDDO-Me, an oleanane synthetic triterpenoid in preventing the progression of preneoplastic lesions to the adenocarcinoma of the prostate in TRAMP mouse model. The growth inhibitory and apoptosis-inducing activity of CDDO-Me contributed to the chemoprevention of prostate tumorigenesis. Furthermore, prosurvival Akt/NF-κB/mTOR signaling pathway appears to mediate the chemopreventive activity of CDDO-Me. Thus, our mechanism based preclinical study provides support for clinical trial of CDDO-Me for chemoprevention of prostate cancer.

## Figures and Tables

**Table 1.** Effect of long-term treatment with CDDO-Me on the incidence and distribution of pathologic lesions in the prostatic lobes.

**Prostate lobe**	**Pre-neoplastic lesions**			**Adenocarcinoma**		
	**Normal**	**LG-PIN**	**HG-PIN**	**WD**	**MD**	**PD**

**DLP**						
Control	0/14 (0%)	0/14 (0%)	4/14 (29%)	9/14 (64%)	1/14 (7%)	0/14 (0%)
CDDO-Me	0/14 (0%)	3/14 (22%)	7/14 (50%)	4/14 (29%)	0/14 (0%)	0/14 (0%)
**VP**						
Control	0/14 (0%)	0/14 (0%)	5/14 (36%)	8/14 (57%)	1/14 (7%)	0/14 (0%)
CDDO-Me	1/14 (7%)	5/14 (36%)	6/14 (43%)	1/14 (7%)	0/14 (0%)	1/14 (7%)

DLP: dorsolateral prostate;VP: ventral prostate;LG-PINL: low-grade PIN;HG-PIN: high-grade PIN; WD: well differentiated;MD: moderately differentiated;PD: poorly differentiated
